# Time-Dependent Changes in Morphostructural Properties and Relative Abundances of Contributors in *Pleurotus ostreatus*/*Pseudomonas alcaliphila* Mixed Biofilms

**DOI:** 10.3389/fmicb.2019.01819

**Published:** 2019-08-09

**Authors:** Silvia Crognale, Silvia Rita Stazi, Andrea Firrincieli, Lorena Pesciaroli, Stefano Fedi, Maurizio Petruccioli, Alessandro D’Annibale

**Affiliations:** ^1^Department for Innovation in Biological, Agro-Food and Forest Systems (DIBAF), University of Tuscia, Viterbo, Italy; ^2^Department of Pharmacy and Biotechnology, University of Bologna, Bologna, Italy

**Keywords:** dual biofilms, *Pleurotus ostreatus*, *Pseudomonas alcaliphila*, microscopy, ultrastructure, biomass estimation, Calgary biofilm device

## Abstract

*Pleurotus ostreatus* dual biofilms with bacteria are known to be involved in rock phosphate solubilization, endophytic colonization, and even in nitrogen fixation. Despite these relevant implications, no information is currently available on the architecture of *P. ostreatus*-based dual biofilms. In addition to this, there is a limited amount of information regarding the estimation of the temporal changes in the relative abundances of the partners in such binary systems. To address these issues, a dual biofilm model system with this fungus was prepared by using *Pseudomonas alcaliphila* 34 as the bacterial partner due to its very fast biofilm-forming ability. The application of the bacterial inoculum to already settled fungal biofilm on a polystyrene surface coated with hydroxyapatite was the most efficient approach to the production of the mixed system the ultrastructure of which was investigated by a multi-microscopy approach. Transmission electron microscopy analysis showed that the adhesion of bacterial cells onto the mycelial cell wall appeared to be mediated by the presence of an abundant layer of extracellular matrix (ECM). Scanning electron microscopy analysis showed that ECM filaments of bacterial origin formed initially a reticular structure that assumed a tabular semblance after 72 h, thus overshadowing the underlying mycelial network. Across the thickness of the mixed biofilms, the presence of an extensive network of channels with large aggregates of viable bacteria located on the edges of their lumina was found by confocal laser scanning microscopy; on the outermost biofilm layer, a significant fraction of dead bacterial cells was evident. Albeit with tangible differences, similar results regarding the estimation of the temporal shifts in the relative abundances of the two partners were obtained by two independent methods, the former relying on qPCR targeting of 16S and 18S rRNA genes and the latter on ester-linked fatty acid methyl esters analysis.

## Introduction

Biofilms are microbial communities attached to a surface and encased within a self-produced matrix generally referred to as extracellular polymeric substances (EPSs). Biofilm formation is one of the most widespread growth strategies of microbiota in natural ecosystems (Branda et al., 2005; Harding et al., 2009; Boudarel et al., 2018). This growth mode confers its cell constituents a higher resistance to shear forces desiccation, and grazing than planktonic cells (McKew et al., 2011; Lemos et al., 2015). Furthermore, one of the most recurring features of biofilm-associated cells is a high tolerance to antimicrobial agents, such as antibiotics, disinfectants, and biocides, which makes biofilms a major problem in clinical and industrial settings (Finkel and Mitchell, 2011; Wolfmeier et al., 2017). These properties have beneficial aspects in bioremediation, where biofilms were often more efficient than planktonic counterparts (Singh et al., 2006; Seneviratne et al., 2008; Pesciaroli et al., 2013a,c; Herath et al., 2014).

Currently, one of the most widely used methods for testing the susceptibility of microbial biofilm to toxic compounds is the Calgary Biofilm Device, also referred to as the MBEC™ device (Ceri et al., 1999). This system consists of a polystyrene lid with 96 downward-protruding pegs that can be adapted to either a standard 96-well microtiter plate or a multichannel trough tray. It has been mainly used for the study of bacterial and yeast biofilms (Ceri et al., 1999; Parahitiyawa et al., 2006; Rivardo et al., 2009; Santopolo et al., 2012). With few exceptions, the system was also used to develop biofilms with filamentous fungi, such as *Aspergillus fumigatus* (Pierce et al., 2008) and, more recently, with the white-rot basidiomycete *Pleurotus ostreatus* (Pesciaroli et al., 2013a,b). Albeit obtained with different approaches, mixed biofilms of this basidiomycete with bacteria have been shown to have a variety of applicative implications, such as endophytic colonization (Jayasinghearachchi and Seneviratne, 2006a) and rock phosphate solubilization (Jayasinghearachchi and Seneviratne, 2006b). Noteworthy, N_2_ fixation by the diazotroph *Bradyrhizobium elkanii* was boosted by its symbiotic association with *P. ostreatus* in a mixed biofilm (Jayasinghearachchi and Seneviratne, 2004). Despite the importance of these *P. ostreatus*-based mixed biofilms, no information is currently available on their architecture. Moreover, the large majority of studies dealing with dual fungal/bacterial biofilm systems have relied on the use of dimorphic fungi, such as *Candida* species (Peters et al., 2010; de Rossi et al., 2014; Sztajer et al., 2014), zygomycetes (Hover et al., 2016), or ectomycorrhizal species (Guennoc et al., 2017, 2018), as the fungal partners.

Thus, to fill this gap, the main objectives of this study were to investigate the time-dependent changes in the morphostructural characteristics of a *P. ostreatus*-based biofilm with a bacterial species and to attempt an estimate of the relative contributions given by each partner in the mixed system. In single-species biofilms, morphostructural changes are often related to biofilm developmental stages, such as maturation, detachment, and transport of nutrients. For instance, void formation and seeding dispersal occur simultaneously in *Pseudomonas aeruginosa* and *Staphylococcus* sp. as the mechanism to promote biofilm maturation and dissemination in response to various stressors (Hunt et al., 2004; Goodwine et al., 2019), while a network of channels possibly involved in the enhancement of nutrients and gas exchange has been observed in *Bacillus subtilis* biofilms (Wilking et al., 2013; Gingichashvili et al., 2017). In multi-species biofilms, the connection between structure and function is less evident although spatial relationships between microbes belonging to different species seem to play an important role in cooperation, exploitation, and competition mechanisms (Liu et al., 2016). For instance, in *Staphylococcus aureus* biofilms, grown in association with Gram-negative bacteria, a competitive rather than cooperative type interaction was observed (Makovcova et al., 2017). Spatial relationships in multi-species biofilms depend on physical properties of cells, such as shape, elasticity, and friction which, in turn, affect major structural features of the biofilm (e.g., thickness, roughness, and cellular alignment) (Farrell et al., 2017; Smith et al., 2017). In the present study, the *P. ostreatus* ATCC 58052 and the *Pseudomonas alcaliphila* 34 strains were selected as the fungal and bacterial partner and mixed biofilm formation was conducted in the MBEC™ system mentioned above. The bacterial strain was selected for this purpose due to its ability to rapidly form biofilms (Santopolo et al., 2012, 2013). An additional reason stemmed from the reported versatility of this species in the degradation of organic pollutants (Ridl et al., 2018), a property that might be useful in the development of mixed systems with *P. ostreatus* which is a widely used species in mycoremediation applications (Leonardi et al., 2008; Covino et al., 2016; Křesinová et al., 2018). The time-dependent changes in the morphostructural properties of the mixed biofilms were investigated by a multi-microscopy approach, including scanning electron, transmission electron, and confocal laser scanning microscopies. Moreover, efforts were devoted to the estimation of the temporal changes in the relative abundances of each partner in the mixed biofilm system. This objective was pursued by two alternative methods relying either on genetic or chemical markers.

## Materials and Methods

### Materials and Growth Media

The MBEC Physiology and Genetics assay device with 96 hydroxyapatite-coated pegs (MBEC™ HA-P&G) combined with conventional 96-well plates (MBEC BioProducts Inc., Edmonton, Canada) was used to develop fungal, bacterial, and mixed biofilms. The growth medium used to develop fungal-bacterial biofilm (FBB) was the low-salt Luria-Bertani (LB) one with the following composition (g l^−1^): tryptone, 10.0; yeast extract, 5.0; NaCl, 5.0.

### Microorganisms

*Pleurotus ostreatus* (Jacquin: Fr.) Kummer, strain ATCC 58052 was stored at 4°C and periodically transferred on malt extract agar (MEA). The *Pseudomonas alcaliphila* B4 strain was a gift of Prof. L. Giovannetti (University of Florence, Italy). The strain, initially stored at −80°C in cryogenic stock, was maintained and periodically transferred on LB agar slants.

### Plate Inhibition Assays

Five-day-old and 2-day-old *P. ostreatus* and *P. alcaliphila* cultures, respectively, grown on LB agar were withdrawn with a sterile loop and concomitantly applied to a 90-mm plate containing the same medium at a distance of 20-mm each one another. The plates were then incubated in the dark at 30°C for 120 h and monitored on a daily basis by visual inspections.

### *P. alcaliphila* Biofilm Formation

Bacterial biofilms (BBs) were grown on the MBEC™ HA-P&G device as described by the manufacturer. In particular, from the cryogenic stock preserved at −80°C, the bacterial strain was spread twice on an LB agar plate. A fresh second sub-culture was used to prepare an inoculum matching a 1.0 McFarland standard (i.e., 3.0 × 10^8^ cfu ml^−1^) as described by the manufacturer (MBEC BioProducts). This suspension was diluted 30 fold with low-salt LB before its addition (150 μL) to each well. The plate was incubated at 30°C under orbital shaking (150 rpm) for 24, 48, and 72 h. After this incubation time, bacterial biofilms were rinsed three times with phosphate-buffered saline (PBS) to remove planktonic and loosely attached cells. Pegs were then broken off with flamed pliers and placed in labeled 2-ml Eppendorf tubes with sterile PBS and biofilm recovered by sonication for 15 min in an Ultrasonic 220 bath (Bransonic, USA) to remove the biofilm off the pegs. The detached biofilm suspensions were recovered by centrifugation at 8.000× *g* for 20 min and the biofilm pellet preserved at −20°C.

### *P. ostreatus* Biofilm Formation

To obtain fungal biofilm (FB), each well of MBEC™ HA-P&G device was loaded with 150 μl of low-salt LB medium and the fungal inoculum (30 μl) was applied by using a multichannel pipette with modified tips, as reported by Pesciaroli et al. (2013b). The inoculated plate was then incubated at 30°C for 4 days under orbital shaking (150 rpm). At the end of incubation, planktonic and loosely adherent biomass was removed by washing each peg with 180 μl of PBS. The washing procedure with PBS was repeated three times. When needed, the fungal biofilm was detached from each peg with the aid of a flexible cell scraper (Sarstedt, Newton, NC, USA) and preserved at −20°C.

### Fungal-Bacterial Biofilm Formation

Fungal-bacterial biofilm (FBB) cultures were obtained applying bacterial inoculum directly on settled *P. ostreatus* biofilms grown for 4 days as reported above. The plate was incubated at 30°C under orbital shaking (150 rpm) and FBB formation on each peg monitored after 24, 48, and 72 h from the bacterial inoculation. After cultivation, the mixed biofilms were rinsed three times with PBS and recovered as described above for mono-specific fungal biofilms.

### Microscopic Analysis of Biofilms

#### Scanning Electron Microscopy

The morphostructural characteristics of FBB biofilms were determined by scanning electron microscope (SEM) analysis. Three replicate pegs with the attached biofilm grown for either 24, 48, and 72 h, were detached with the aid of a plier and prepared as reported by Pesciaroli et al. (2013b). The observation of samples was made by a JSM 6010 LA analytical SEM featuring integrated energy dispersive spectroscopy (EDS; JEOL, Tokyo, Japan).

#### Transmission Electron Microscopy

Samples of mixed biofilms were fixed and dehydrated as above and then infiltrated for 3 days with decreasing ethanol/LR White resin (SPI Supplies, West Chester, PA) ratios. At the end of the procedure, samples were embedded in LR White resin and cut with a Reichert Ultracut ultramicrotome (Leica Microsystems Srl, Milan, Italy) using a diamond knife. Thin sections (60–80 nm) were collected on copper grids, stained with uranyl acetate and lead citrate, and observed with a JEOL 1200 EX II electron microscope (JEOL, Tokyo Japan). Micrographs were acquired by the Olympus SIS VELETA CCD camera equipped with the iTEM software package (Olympus Soft Imaging Solutions GmbH, Münster, D).

#### Confocal Laser Scanning Microscopy

Mixed biofilm samples were washed with saline solution for 1 min under orbital shaking (100 rpm). Then, each biofilm was stained for 30 min at 30°C with a LIVE/DEAD *Bac*Light bacterial viability kit (Molecular Probes, Inc., Eugene, OR) in 0.9% NaCl solution. Briefly, 1 μl of a mixed solution containing 1.86 mM SYTO-9 and 2.72 mM propidium iodide (PI) was added to 179 μl of 0.9% NaCl and shaken as above. Each peg was then washed in 0.9% NaCl solution for 1 min, dipped for 15 min with 0.02% (w/v) Calcofluor White Stain M2R (CFW; Fluka) in 0.9% NaCl, and then washed as above. Samples were observed with an LSM 710 confocal scanning laser microscope (Zeiss, Oberkochen, D). Confocal images of green (SYTO-9; Ex/Em, 480/500 nm), red (PI; Ex/Em, 490/635 nm), and blue (CFW; Ex/Em, 405/450 nm) fluorescence were acquired simultaneously using a multi-track mode. A three-dimensional model of the biofilm was computed using the ZEN 2012 lite software package (Zeiss). Mixed biofilms were also stained with FUN-1 viability probe (Molecular Probes, Inc.).

### DNA Extraction and Quantification

Total genomic DNA was isolated from freeze-dried biofilm by using the PowerSoil® DNA Isolation Kit (MoBio Laboratories, Carlsbad, CA) following the manufacturer’s instructions. Extracted DNA, analyzed by agarose gel (1% w/v) electrophoresis followed by staining with ethidium bromide, was photographed under UV transillumination with a GelDoc XR digital camera (Bio-Rad, USA) and quantified using a Qubit Fluorometer (Invitrogen).

### Quantitative PCR Analysis

The 16S and 18S rRNA genes were used as target sequences for amplification of bacterial and fungal component, respectively. In particular, 16S rDNA sequences were amplified from either mono-specific bacterial or mixed biofilms with the primer pair 331F (5′-TCCTACGGGAGGCAGCAGT-3′) and 797R (5′-GGACTACCAGGGTATCTAATCCTGTT-3′) (Nadkarni et al., 2002). Instead, 18S rDNA sequences were amplified from either mono-specific fungal or mixed biofilms with the primer pair FR1F (5′-AICCATTCAATCGGTAIT-3′) and FF 390R (5-′CGATAACGAACGAGACCT-3′) (Vainio and Hantula, 2000). Real-time PCR assays were carried out on the LightCycler® 480 System (Roche Applied Science, USA). The quantification was based on the SYBR-green fluorescent dye, which was bound to double-stranded DNA during PCR amplification. Each reaction was performed in a 20-μl volume containing 5 μl of DNA template, 1 μl of each primer, and 10 μl of SYBR Green PCR Master Mix (Roche, Milan-Italy). The conditions used for 16S gene amplification were those reported by Nadkarni et al. (2002). The fungal amplification conditions, instead, were those reported by Vainio and Hantula (2000) except for a reduced primer concentration (0.125 μM vs. 0.250 μM) and a slightly increased annealing temperature (53°C vs. 50°C). Melting curve analysis of the PCR products was conducted following each assay to confirm that the fluorescence signals were due to specific PCR products and not to other artifacts, such as, for instance, primer-dimer formation. In mixed biofilms, the DNA concentrations of *P. ostreatu*s and *P. alcaliphila* were calculated based on standard curves obtained using 10-fold serial dilutions of a known concentration of plasmid pGem T-easy cloning vector containing the target regions and, finally, expressed as 18S and 16S rRNA gene copy number, respectively. For a better understanding and comparison of the results with other methods, calibration curves, based on linear regression models, were constructed for mono-specific biofilms to correlate copy numbers of target genes to biofilm’s biomass dry weights. Basically, gDNA was extracted, as described above, from different amounts of each lyophilized mono-specific biofilm (0–10 mg) and samples were further analyzed by q-PCR. The gene copy numbers thus obtained were plotted against the respective biomass amounts for each biofilm system and data fitted by linear regression.

### Ester-Linked Fatty Acid Methyl Ester Analysis

Identification and subsequent quantification of fungal and bacterial fatty acids were performed on lyophilized cells by a modified alkaline methanolysis method (Schutter and Dick, 2000). In brief, either freeze-dried mono-specific or mixed biofilms (25.0 ± 0.2 mg dry weight) were added with 15 ml of a 0.2 M KOH methanolic solution in screw-cap centrifuge glass tubes (50 ml capacity). Then, the reaction mixture was added with 0.1 ml of methyl-nonadecanoate (0.23 mg ml^−1^ in hexane), as the internal standard, and then incubated for 1 h at 37°C with periodical vortexing. At the end of the incubation, the pH was neutralized by adding 6.6 ml of 1.0 M acetic acid in double-distilled water. The mixture was added with 20-ml n-hexane, mixed repeatedly by gentle inversion, and centrifuged (800× *g*; 8 min). After removal of the upper organic phase, a fresh aliquot of hexane (20 ml) was added, mixed, and centrifuged again as above. The pooled organic phases were transferred to a vacuum flask and the organic phase removed in an R-210 Rotavapor (Büchi Labortechnik, Flawil, CH) at 30°C. The dried residue was then dissolved again in n-hexane (1.0 ml) before gas-chromatography mass spectrometry analysis, which was performed as described in Stazi et al. (2017). Mass spectra were recorded by the use of a QP-5050 (Shimadzu, Japan) spectrometer and methylated fatty acids were identified according to their mass spectra and using BAME 24 and 37-Component FAME Mix (47080-U and 47,885-U, respectively, Sigma-Aldrich, Milan, Italy) as the chemical standards. The polyunsaturated fatty acids 18:2ω6,9 and cy17 were used as signature markers for fungal and bacterial biomass since their relative percent abundances to the total sum of fatty acid methyl ester (FAME) remained constant in mono-specific fungal and bacterial biofilms in the time intervals considered in this study (96–168 and 24–72 h, respectively). Thus, fungal and bacterial biomasses in biofilms were calculated from the following expression:

$$\mathrm{Biomass}\left(\mathrm{mg}\right)=\frac{C_{MXB_{t}}\cdot \;W_{MXB_{t}}}{C_{MB_{t}}}$$

where C_MBt_ and C_MXBt_ are the marker concentrations at time t, expressed in μmoles g^−1^ biofilm dry weight, in coeval mixed and mono-specific biofilms, respectively, and W_MXBt_ is the weight of the mixed biofilm. The bacterial and fungal biomasses thus estimated were added and referred to the total sum of biomasses to calculate their relative percent contribution.

### Statistical Analysis

Statistical analysis of data and pair-wise comparisons were performed by using the SigmaStat software package (Jandel Scientific, Germany).

## Results

### Mixed Biofilm Formation and Morphostructural Analysis

**Figure 1:**
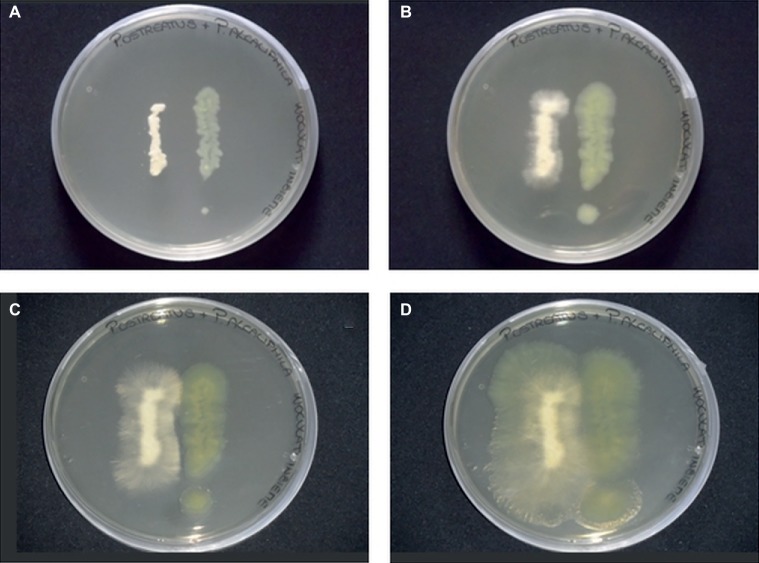
Plate inhibition assays of *P. ostreatus* (left side) and *P. alcaliphila* (right side) grown on Luria Bertani agar (1.5%) for 24 **(A)**, 48 **(B)**, 72 **(C)**, and 120 **(D)** h at 30°C in the dark.

In the present study, agar plate assays, conducted by co-culturing *P. alcaliphila* and *P. ostreatus*, indicated the absence of significant mutual inhibition phenomena. Neither the occurrence of inhibition halos around colonies nor the tendency of the growing fronts of the two partners to preferentially proceed in opposite directions was observed (Figure 1). However, upon incubation, due to its markedly shorter generation times, *P. alcaliphila* tended to colonize even the surface where the mycelium had previously grown while *P. ostreatus* was unable to occupy plate sections where bacterial growth had taken place (Figure 1D).

Based on these results and in the attempt at simulating natural systems where the basidiomycetes offer a hyphal network for adhesion and movement of soil bacteria (Deveau et al., 2018; Guennoc et al., 2018), the application of bacterial inocula to already settled fungal biofilms was deemed to be the best approach to mixed biofilm formation. The application of the bacterial inoculum was done 4 days after the fungal inoculation since this time interval was found to enable the formation of *P. ostreatus* biofilms that, besides being stable (Pesciaroli et al., 2013b), met the structural requirements for fungal biofilms set by Harding et al. (2009).

**Figure 2:**
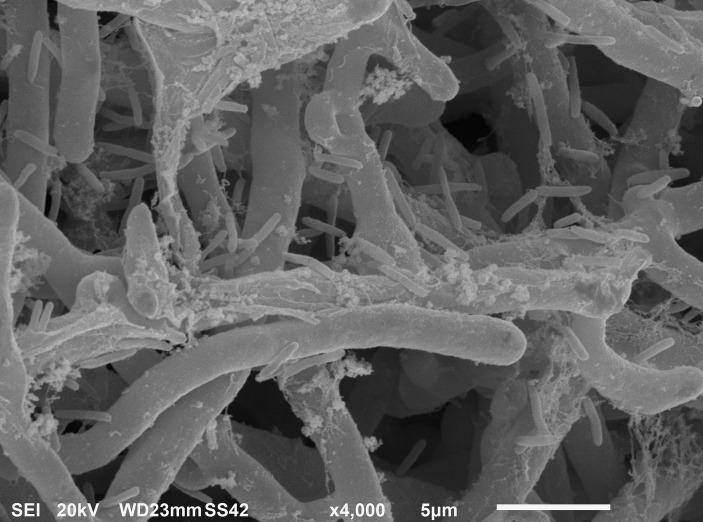
Scanning electron micrograph showing *P. alcaliphila cells* attached to 4-day-old *P. ostreatus* biofilm after 6 h from bacterial inoculation at a 4,000× magnification.

**Figure 3:**
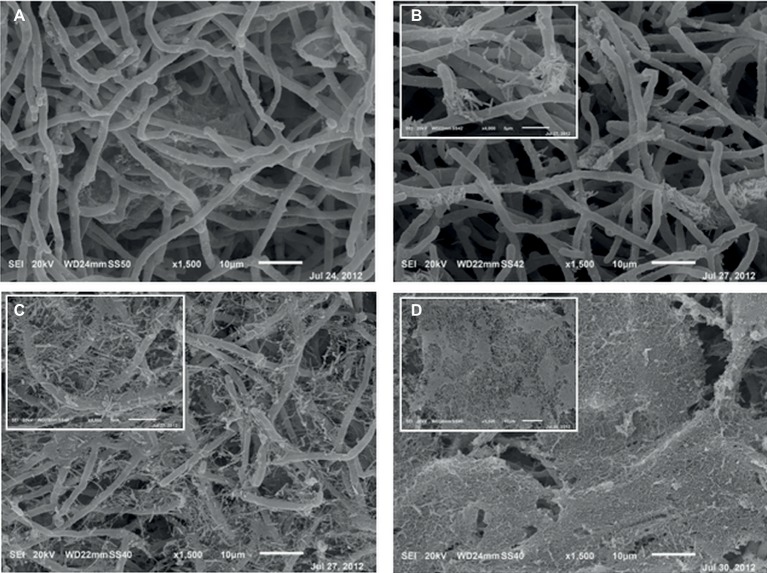
Scanning electron micrographs of 4-day-old mono-specific *P. ostreatus* biofilm **(A)** and its mixed biofilms with *P. alcaliphila* obtained after 24 **(B)**, 48 **(C)**, and 72 **(D)** h of incubation at 30°C under orbital shaking (150 rpm) on MBEC™ HA-P&G system. All micrographs are at a 1,500× magnification while insets in **(B)** and **(C)** at higher magnification (4,000×). Inset in **(D)** shows the bacterial biofilm grown in the proximity of the air-liquid interface (1,500×).

After 6 h from the application of the bacterial inoculum to the established fungal biofilm, the presence of mostly isolated cells or less frequently of aggregated cells *P. alcaliphila* adhering on the surface-attached mycelium was evident, as shown in Figure 2, and the adhesion appeared to be mediated by the presence of either filaments or aggregates of ECM. As a matter of fact, before inoculation with *P. alcaliphila*, 4-day-old mono-specific biofilms of *P. ostreatus* were characterized by a highly intertwined hyphal network with a generally smooth surface interspersed sporadically with the presence of filaments of ECM, as shown in Figure 3A. A reticular structure of ECM filaments of bacterial origin was evident after 48 h from inoculation (Figure 3C and inset) and it tended to assume a tabular semblance after 72 h that overshadowed partially the underlying mycelial network (Figure 3D). Irrespective of the incubation time, the development of the attached mycelium to the hydroxyapatite-coated polystyrene surface of the peg took place only on the distal length (i.e., 3.0–5.0 mm) close to the peg’s tip. Conversely, adherent bacterial cells tended also to fully colonize peg areas located above the air-liquid interface, which was not forced instead, by the attached mycelium (Figure 3D, inset). Thus, these observations indicated clearly a time-dependent increase in the relative contribution of the bacterial partner to the makeup of the biofilm in the available peg’s surface.

**Figure 4:**
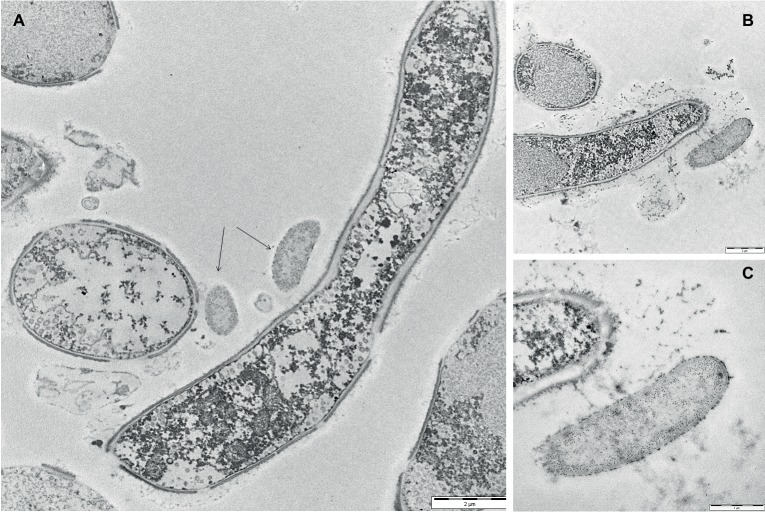
Transmission electron micrographs of 24-h-old mixed *P. ostreatus*-*P. alcaliphila* biofilms. **(A)** Longitudinal and cross-view sections of *P. ostreatus* hyphae and adherent *P. alcaliphila* cells (see black arrows) (length of the bar: 2 μm). **(B)** The longitudinal section of the terminal portion of a *P. ostreatus* hypha with EPS-promoted attachment of a bacterial cell (length of the bar: 2 μm). **(C)** Magnification of **(B)** at the hyphal tip (length of the bar: 1 μm).

To gain insights into the initial adhesion mechanisms of bacterial cells on the pre-formed adherent mycelial network, mixed biofilm samples were analyzed by TEM after 24 h from the application of the bacterial inoculum. Figure 4A, showing both cross-view and longitudinal sections of *P. ostreatus* hyphae, confirmed that the adhesion of bacterial cells onto the mycelial cell wall appeared to be mediated by the presence of an abundant layer of ECM. The same observation was done close to a hyphal tip where ECM production was rather abundant (Figure 4B). Figure 4C, showing a bacterial cell in the immediate proximity of the hyphal tip, besides highlighting the presence of a thick ECM layer lining both the bacterial cell and the hyphal tip, also shows the presence of filamentous pili-like structures.

**Figure 5:**
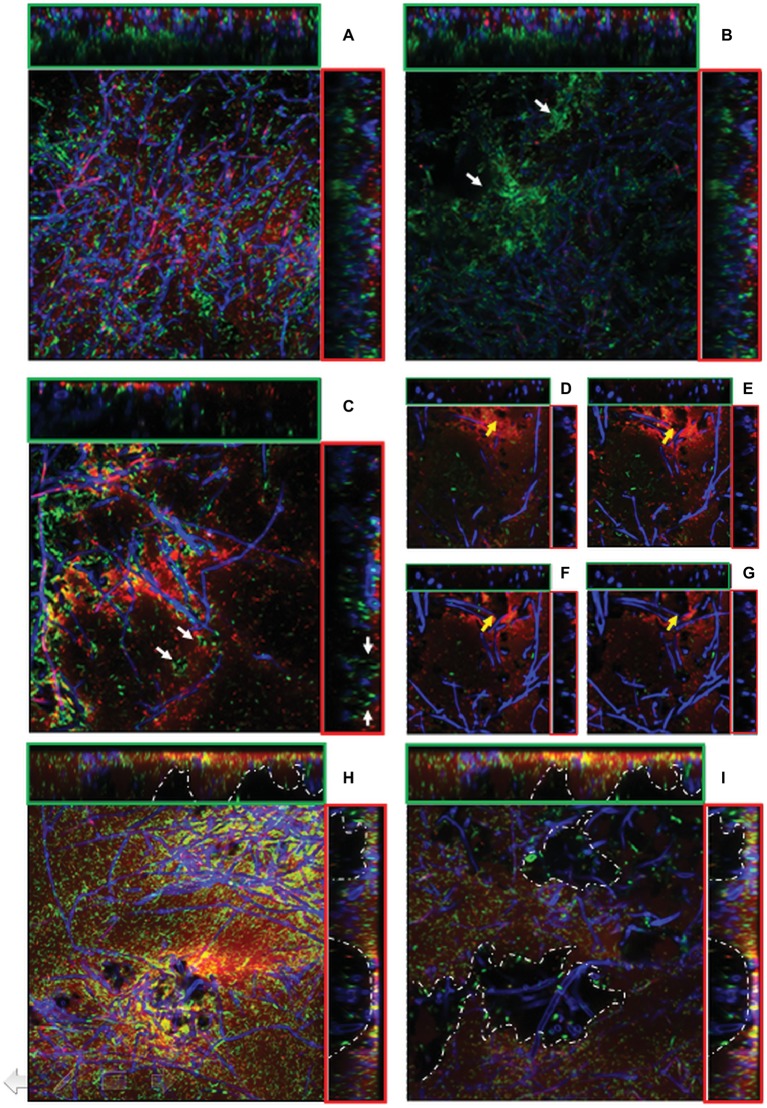
**(A**–**I)**. CLSM images of mixed *P. ostreatus-P. alcaliphila* biofilm after 24 h **(A**,**B)**, 48 h **(C**–**G)**, and 72 h **(H**–**I)** from bacterial inoculum application after concomitant Syto 9 (green fluorescence) and Propidium iodide (red fluorescence) stain followed by Calcofluor staining. Orthogonal y-z and x-z views are delimited by green and red frames, respectively. Each image represents an area of 45.17 × 10^3^ μm^2^ (magnification 40×) and 18.21 × 10^3^ μm^2^ (magnification 63×). **(A)** sub-population of non-clustered viable bacterial cells in 24-h-old mixed biofilm (x-y view) with initial ECM deposition in the outer layer (x-z and y-z orthogonal views); **(B)** viable bacterial cluster in close proximity of inner voids (x-y view, white arrows) in 24-h-old mixed biofilm; **(C)** localization of viable bacterial cells at the edges of voids in 48-h-old mixed biofilm (x-y view, white arrows) (40× magnification);. **(D–G)**, Increase in the lumina of void channels in 48-h-old biofilms from a depth of 1.03–4.24 μm (x-y view, yellow arrows) (63× magnification); **(H)** 72-h-old mixed biofilm characterized by a compact structure with interspersed void channels indicated by white arrows (depth: 6.43 μm, magnification: 40×); **(I)** the edges of large cavities are delimited by a dotted white line in 72-h-old biofilms (depth: 23.54 μm, magnification: 40×).

CLSM showed that the firmly attached mycelial network supplied a loose scaffold which was gradually filled by either bacterial cells and/or ECM of bacterial origin as the incubation time increased. As already observed by SEM, in the outer layers of 24-h-old mixed biofilms, bacteria tended to adhere on the hyphal surface predominantly as isolated cells rather than in a clustered mode and an initial deposition of ECM was evident in the outer layers (Figure 5A, x-z and y-z orthogonal views). SYTO-9/PI staining showed that similar amounts of viable and dead cells were visible in the outer layers (5.35 μm depth). Conversely, in the inner layers (23.54 μm depth), the large majority of bacteria were viable and tended to cluster together nearby of void spaces and in association with the hyphal surface, leading to a highly compacted structure of such microcolonies (Figure 5B). After 48 h, regardless of the biofilm depth, an increased density of adherent bacterial cells onto the fungal mycelium was evident. This was associated with the presence of abundant ECM deposition as made evident from an intense red fluorescence due to PI binding onto nucleic acids, which are known ECM components in both fungal (Mitchell et al., 2016) and bacterial biofilms (Domenech et al., 2016; Ibáñez de Aldecoa et al., 2017) (Figures 5C–G). In another type of graphical representation, referred to as Volume Rendering, a 3D reconstruction computed from two-dimensional images stack shows that bacterial cells and ECM deposition were mostly located close to the mycelium (Supplementary Figures S1A–D). Microcolonies of viable bacterial cells tended also to locate along the edges of voids (Figure 5C). Also, a depth-dependent increase in the lumina of voids was evident (Figures 5D–G).

In 72-h-old mixed biofilms, and within a 5- to 25-μm depth range, the overall structure was significantly more compact than that observed at previous sampling times with a notable increase of bacterial cells encased in an abundant ECM (Figure 5H). Moreover, compared to 24- and 48-h-old mixed biofilms, increased frequency of voids and respective diameters of lumina were observed (Figures 5H,I). This increase in the lumina of void channels brought to the formation of cavities in the inner layers characterized by a loose biofilm structure and by a non-homogeneous ECM distribution (Figure 5I, x-z, and y-z orthogonal views). At a depth higher than 30 μm, where the overall biofilm organization had a heterogeneous architecture characterized by the presence of loose and lacunose structure, a neat increase in the relative proportion of viable bacterial cells, mainly localized in correspondence of these cavities, was observed (Figure 5I).

### Estimation of the Relative Abundances of Bacterial and Fungal Partners in Mixed Biofilms

#### q-PCR-Based Approach

In order to quantify the time-dependent changes in the relative contribution of each partner to mixed biofilms, a molecular method relying on q-PCR targeting of 16S and 18S rRNA genes was used. From preliminary trials, it was found that the Power Soil extraction kit besides ensuring a high reproducibility also yielded genomic DNA from both *P. alcaliphila* and *P. ostreatus* with a high degree of purity. However, a different DNA extraction yield from *P. alcaliphila* and *P. ostreatus* (from 13.2:1.0 to 21.0:1.0) was observed, probably due to the different composition of their cell wall, but also to the different ratio between total biomass and DNA content within the two species.

**Figure 6:**
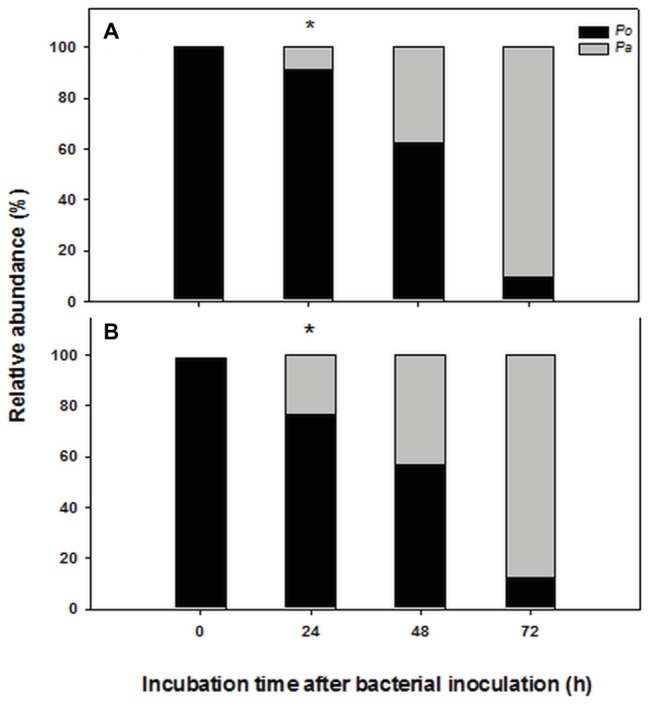
Time-dependent changes in percent contribution of fungal (black) and bacterial (gray) biomass in mixed biofilms obtained by inoculating *P. alcaliphila* (*Pa*) on already established 4-day-old *P. ostreatus* (*Po*) biofilms and incubating for 24, 48, and 72 h at 30°C under orbital shaking (150 rpm). Plot **(A)** refers to the estimates obtained by q-PCR analysis of 16S and 18S rRNA genes, respectively while plot **(B)** refers to those obtained by ester-linked fatty acid methyl esters analysis. Pair-wise comparison between estimates obtained at the same incubation time with the two methods was performed by the Student’s *t*-test after arcsin of the square-root transformation of percent data. The presence of an asterisk above bars denotes the presence of statistically significant differences.

The chosen primer pairs employed for q-PCR experiments for either *P. alcaliphila* or *P. ostreatus* were found to be highly adequate for the purpose, since they did not either give rise to the formation of unspecific products, such as primer-dimer formation, or cross-amplification. Supplementary Figures S2A,B show the calibration curves relating fungal and bacterial target genes from mono-specific biofilms to respective threshold cycles (C_t_). The values of the coefficients of determination (R^2^) for both regressions were 0.99. On the basis of these curves, it was possible to determine the amounts of fungal and bacterial DNA in terms of 18S rDNA and 16S rDNA copy number respectively, within mixed biofilms as a function of incubation time. Table 1 shows that bacterial DNA content in mixed biofilms started to increase after 24 h of incubation reaching its maximum after 72 h where its concentration was found to be two orders of magnitude higher than that related to the bacterial inoculation time (time zero). Conversely, a slight decrease in fungal DNA was observed after 24 h. The relationships between gene copy number and biomass dry weight in the bacterial and fungal mono-specific biofilms were analyzed by a linear regression model (Supplementary Figure S3) and the respective slopes of the regression lines used to estimate the relative biomass contributions of each partner to the mixed biofilm. Figure 6A shows clearly that the relative contribution of the fungus markedly declined from 91.2 to around 9.5% as the incubation time was extended from 24 to 72 h.

**Table 1:** q-PCR quantification of bacterial (B) and fungal (F) gene copy number in mixed biofilm and their relative ratios at different incubation times.

Time (h)	16S RNA gene (copy number mg^−1^ biomass dry weight)	18S RNA gene (copy number mg^−1^ biomass dry weight)	F/B
0	9.38 × 10^6^	2.85 × 10^8^	30.40
24	2.88 × 10^8^	2.62 × 10^8^	0.91
48	4.18 × 10^8^	5.56 × 10^7^	0.13
72	7.02 × 10^9^	5.92 × 10^7^	0.01

#### Ester-Linked Fatty Acid Methyl Ester-Based Approach

Keeping in mind the adopted conditions for mixed biofilm formation, the FAME profiles were analyzed in mono-specific biofilms that had been grown in the 0- to 72-h (*P. alcaliphila*) and 96- to 168-h (*P. ostreatus*) time intervals and Table 2 shows their average FAME compositions within the intervals mentioned above. The most abundant FAMEs in *P. alcaliphila* sp. biofilms were 18:1ω9(t + c), 16:0, and cy17 while in *P. ostreatus* biofilms, 18:2ω6,9 and 16:0 were the predominant components. Irrespective of their concentrations in the respective biofilms, several FAMEs were shared in the two biofilms as shown in Table 2. Significant exceptions were cy17, 18:1ω9(t + c) and 18:0 which were detected only in the *P. alcaliphila* biofilms while 18:2ω6,9 and 17:1 only in *P. ostreatus* ones. Among these, however, only cy17 and 18:2ω6,9 maintained constant relative abundances with respect to total FAME since their coefficients of variation amounted to 0.031 and 0.046, respectively, throughout the time intervals under study. For this reason, the former and the latter were used to estimate bacterial and fungal biomass, respectively, in mixed biofilms. Figure 6B shows that the estimates, relying on chemical markers, did not differ markedly from those obtained by q-PCR except those related to mixed biofilms that had been incubated for 24 h after bacterial inoculation (77.5 vs. 91.5%). At the end of the experiment, namely after 72 h from bacterial inoculation, the relative abundance of *P. ostreatus* in the mixed biofilm was estimated to amount to 12.5 ± 1.8%. It is evident that especially in 48- and 72-h-old biofilms, the sum of the biomasses of the two partners did not explain the whole biofilm mass, due to the production of ECM, which is inherent to biofilm systems.

**Table 2:** Ester-linked fatty acid methyl ester (El-FAME) analysis in mono-specific *P. alcaliphila* and *P. ostreatus* biofilms.

Retention time (min)	FAME	*P. alcaliphila* (μmoles g^−1^)	*P. ostreatus* (μmoles g^−1^)
19.718	14:0	7.69 ± 0.76	n.d.
21.48	15:0	25.14 ± 1.37	2.29 ± 0.19
22.863	16:1ω7	50.17 ± 21.90	0.51 ± 0.05
23.193	16:0	171.09 ± 5.63	27.63 ± 2.31
24.534	17:1	n.d.	0.40 ± 0.18
24.54	cy 17	57.89 ± 1.77	n.d.
24.727	17:0	21.86 ± 0.67	0.82 ± 0.1
25.875	18:2ω6,9	n.d.	113.53 ± 5.2
25.887	18:1ω9t	4.09 ± 0.63	n.d.
26.025	18:1ω9c	234.95 ± 3.75	0.41 ± 0.05
26.246	18:0	8.31 ± 0.28	n.d.

*Data are the means ± standard error of biofilms grown for 0–72 h and 96–168 h for the former and the latter species, respectively*.

## Discussion

Several types of interaction between *P. ostreatus* and Pseudomonads have been reported yet and found to range from the growth-promoting effect on this fungus by some *Pseudomonas* species (Cho et al., 2003) to antibacterial effects exerted by the fungus toward members belonging to this bacterial genus (Hearst et al., 2009; Bawadekji et al., 2017). Direct involvement of *P. fluorescens* in morphogenetic events in *P. ostreatus*, such as primordia formation and development of basidiomes, was proved (Cho et al., 2003). As a consequence, co-cultivation plate assays were used preliminarily to assess the compatibility between this fungus and *P. alcaliphila*. Although these assays did not show the occurrence of evident mutual inhibition phenomena, the capacity of the bacterial partner to occupy plate sections that had been previously colonized by the other partner was not shared by *P. ostreatus*. The competitive success of a given species depends also on its combined ability to capture the other’s domain and retain its one, a colonization model that has been referred to as secondary resource capture (Holmer and Stenlid, 1993; Woodward and Boddy, 2008). Results obtained in these co-cultivation assays suggest that *P. ostreatus* was unable to act *via* secondary resource capture and, thus, it was not a strong competitor toward *P. alcaliphila*.

For mixed biofilm formation, the Calgary biofilm device was preferred over other alternative approaches since free-floating and loosely bound cells can be easily detached from peg-bound biofilms thus reducing the interference of planktonic cells and due to its previously observed ability to deliver reproducible *P. ostreatus* mono-specific biofilms (Pesciaroli et al., 2013b). To prepare mixed biofilms, bacterial inocula were applied to already settled fungal biofilms based both on the previously mentioned co-cultivation plate assays and on ecological considerations. Filamentous fungi are known to constitute up to 75% of the microbial biomass in the upper layers of soil generating mycelial networks the extension of which is reported to range from 10^2^ to 10^4^ m length per g of topsoil (Ritz and Young, 2004), thus offering vast surfaces for bacterial biofilm settlement (Guennoc et al., 2018) and providing fungal highways for bacterial movement (Banitz et al., 2013).

Although, as already mentioned, a variety of *P. ostreatus*-based binary biofilms with bacteria have been studied concerning their practical applications (Jayasinghearachchi and Seneviratne, 2004, 2006a), there is a total lack of information regarding the structural organization and its dynamics over time in these systems. In the present study, a multi-microscopy approach was adopted to investigate the time-dependent changes in *P. ostreatus*-*P. alcaliphila* mixed biofilms since it is known that the sample dehydration steps before TEM and SEM analysis induce distortion in biofilm architecture (Chandra et al., 2001). As a consequence, although considerable insights into biofilm ultrastructure have been derived by the use of these techniques, the direct examination of intact biofilms, enabled by CLSM, was deemed to be necessary, in agreement with the suggestions of Melloul et al. (2016).

In this study, the CLSM analysis, in addition to showing that the initial phase of adhesion to the hyphal surface preferentially involved isolated bacterial cells and was mediated by filamentous or aggregated ECM structures, in agreement with SEM and TEM observations, also indicated a gradual accumulation of bacterial cells on the mycelial surfaces in the inner biofilm layers. In spite of an observed increase in compactness in the structure of the outer surface layers of the biofilm, there was a noticeable increase in the frequency of empty spaces (voids) and this was also associated with an increase in the diameter of their lumina in the innermost layers. Several studies have claimed the occurrence of both interstitial voids and channels in microbial biofilms (Di Bonaventura et al., 2006; Stewart and Franklin, 2008). In this respect, it has to be noted that the aggregate growth of microorganisms in biofilms decreases the accessibility of nutrients into inner layers and, at the same time, the diffusion of waste products to the external environment and these structural adaptations serve the purpose of counteracting these phenomena (Stewart and Franklin, 2008). Thus, the presence of open channels within the biofilm has been shown to mitigate these internal mass transfer limitations through the creation of a transport system capable of aiding distribution of water, oxygen, and nutrients and disposal of metabolic waste products (Di Bonaventura et al., 2006; Kataky and Knowles, 2018). The placement of bacterial microcolonies on the edge of the voids, which was observed in the present study, could favor them in gas exchange and nutrients acquisition.

In the present study, CLSM analysis also highlighted a differential distribution of bacterial cells across the thickness of the biofilm; in particular, the higher frequency of viable than dead cells, observed in the innermost biofilm layers was also reported in other biofilm systems (Boles and Singh, 2008). This phenomenon has been traced back to the physiological heterogeneity found in biofilms and mainly ascribed to the presence of oxygen and nutrient concentration gradients (Stewart and Franklin, 2008). In particular, cells in the inner layers were less susceptible to oxidative stress than those located in outer layers due to diffusional limitations of oxygen (Boles and Singh, 2008; Ryder et al., 2012). In particular, several studies found that microbial cells populating the outermost biofilm layers, in close contact with the bulk liquid interface, were more susceptible to the accumulation of reactive oxygen species (ROS) and thus to oxidative stress (Stewart and Franklin, 2008; Oh and Jeon, 2014). The time-dependent evolution of ECM from filamentous to reticular and, finally, to tabular semblance, which was found by SEM, was also observed in a dual biofilm system involving the ectomycorrhizal basidiomycete *Laccaria bicolor* and a *Pseudomonas fluorescens* strain (Guennoc et al., 2017).

The estimation of the relative contribution of the two partners in the makeup of a dual biofilm is very important to explain both the structural and functional properties of the system (Lopes et al., 2018). To this aim, the approach of colony counting has been used for fungal quantification in dual biofilms involving dimorphic species, such as *C. glabrata* and *C. albicans* in association with either *Streptococcus mutans* (Pereira-Cenci et al., 2008) or *Staphylococcus aureus* (Zago et al., 2015). However, in vegetative mycelia under non-sporogenic conditions, fungal propagules, potentially able to yield a colony, are characterized by different degrees of hyphal fragmentation the extent of which is susceptible to variation during sampling, dilution, plating, and spreading operations. As a consequence, although CFU count is the most commonly used method for fungal quantification, due to its simplicity, several concerns have been raised about the ability of the method to provide reproducible and reliable estimates of fungal growth at least in filamentous fungi (Pitt, 1984; Schnürer, 1993; Mensah-Attipoe et al., 2016). In a recent study aimed at comparing five different assay methods for the measurement of fungal growth, namely colony counting, determination of ergosterol or beta-N-acetylhexosaminidase activity, quantitative PCR and microscopic spore counting, the first method exhibited a coefficient of variation of replicated samples (CV_r_) that was twice as high the other methods (Mensah-Attipoe et al., 2016). Moreover, marked discrepancies between plate count and molecular tools, relying on qPCR of 16S rRNA and peptide nucleic acid probe fluorescence *in situ* hybridization (PNA-FISH), were even observed in quantifying populations inhabiting dual and three-membered bacterial biofilms (Lopes et al., 2018). The same authors suggested that at least two quantification techniques might be needed to gain reliable estimates of the relative abundances of the biofilm’s contributors. For the above reasons, in the present study, two cultivation-independent methods were comparatively used in the attempt of pursuing this goal. The former method relied on quantitative real-time polymerase chain reaction (qPCR) of 16S and 18S rRNA genes. As far as the choice of the genomic DNA extraction method was concerned, it was taken into account that microbial biofilms are surface-attached heterogeneous communities encased within an abundant extracellular polysaccharide matrix which makes largely difficult this task. Within this frame, several studies point out that extraction protocols designed for soil samples are more performing in extracting DNA from biofilms than those designed for other matrices (Miller et al., 1999; Ferrera et al., 2010). Among available soil extraction protocols, the Power Soil extraction kit was selected in this study since it was previously found to be the most efficient one in extracting genomic DNA from filamentous fungi (Fredricks et al., 2005). In the present study, the primer pair concentrations were optimized and no artifacts, such as primer dimers formation and cross-amplification products, were found as assessed by melting curve analysis. The use of this method highlighted a time-dependent decrease in the relative abundance of *P. ostreatus* in mixed biofilms with *P. alcaliphila*. Noteworthy, a similar approach was used successfully in *C. albicans*/*S. mutans* biofilms (Sztajer et al., 2014) and, more recently, qPCR of 23S and 28S rRNA genes was used to estimate the relative abundances of *Stenotrophomonas maltophilia* and *Aspergillus fumigatus* in dual biofilms (Melloul et al., 2016).

The second quantification method relied on the use of fatty acid methyl esters (FAMEs) as possible markers of fungal and bacterial abundance in the mixed biofilm systems. A similar approach was used as a means of discriminating *P. ostreatus* from bacteria in a variety of dual biofilms (Melucci et al., 2013) and although it succeeded from a qualitative viewpoint, it was unable to estimate the relative abundances of the two partners (Melucci et al., 2013). The EL-FAME method used in the present study is unable to produce FAMEs from free fatty acids and, as a consequence, those detected by this approach derive uniquely from the transesterification reactions (Schutter and Dick, 2000). The presence of lipid esters downstream of the cell lysis is deemed to be highly unlikely due to their extremely rapid turnover rates and, for this reason, FAME-based profiling techniques are believed to deliver a snapshot of active members of microbial communities (Frostegård et al., 2011; Oates et al., 2017). Unique exceptions to this assumption are represented by experiments involving the exposure of the biological system under consideration to toxic substances able to promote concomitant cell death and inhibition of phospholipid-degrading enzymes or cultivation at very low temperatures (Frostegård et al., 2011). Hence, in addition to providing similar estimates to those relying on q-PCR analysis on the relative partner contributions to biofilm makeup, this technique also yielded indirect evidence on the residual viability of *P. ostreatus* that, as opposed to that of *P. alcaliphila*, was not demonstrated *in situ* in the mixed biofilm by CLSM (present study). Thus, albeit the reduced concentration of 18:2 ω6,9 reflected the shift in the relative abundances of the two partners, its presence, even in 72-h-old mixed biofilms, derived from viable mycelium. Indeed, it is known that in some dual biofilms, such as those involving *Candida albicans* and virulent *Pseudomonas aeruginosa* strains, the establishment of the bacterial biofilm over the surface of the fungus might lead to its death (Hogan and Kolter, 2002). In a subsequent study, the ability of *P. aeruginosa* to kill *C. albicans* cells was shown to occur through a process involving the homoserine lactone quorum-sensing molecule, 3-oxo-C12 (Hogan et al., 2004). Moreover, it is known that the close interaction with some bacterial species can be detrimental to fungi; that, as a consequence, produce defensins (Essig et al., 2014), or quorum quenching enzymes (Stöckli et al., 2017) to prevent the colonization of their hyphal surface by bacteria.

## Conclusions

This study provides insights into the dynamic changes in the architecture of a dual biofilm by a multi-microscopy approach where the selected fungal partner, i.e., *P. ostreatus*, has been previously shown to be involved in biofilm systems with important applicative implications. The bacterial partner, which was selected on the basis of its compatibility with the fungus and fast biofilm-forming ability, was capable of using the surface-attached fungal biofilm as a loose scaffold for its settlement and colonized also the inner layers of the biofilm locating on the edges of cavities, the lumina of which increased with biofilm depth. In light of the objective difficulties in estimating the relative abundances of different partners in multi-component biofilms, and despite the presence of significant ECM, the two different approaches used in this study gave similar results.

On an overall basis, the methodologies applied to this study allowed to estimate the single contribution of each species in a mixed fungal/bacterial biofilm and to monitor the distribution of each species in the biofilm architecture.

## Data Availability

The datasets generated for this study are available on request to the corresponding author.

## Author Contributions

MP and AD’A provided funding and conceived the experimental setup. LP and SC performed the preparation and SEM and TEM analyses of biofilms. SC and SS performed biomass estimation by qPCR and el_FAME anaylses, respectively. SF and AF performed CLSM analysis. All of the authors contributed to the preparation of the manuscript.

### Conflict of Interest Statement

The authors declare that the research was conducted in the absence of any commercial or financial relationships that could be construed as a potential conflict of interest.

## Supplementary Material

SUPPLEMENTARY FIGURE S1 Volume rendering of *P. alcaliphila* and *P. ostreatus* 48-h-old mixed biofilm. Image A is the overlay of the SYTO 9 (B, live bacterial cells), PI (C, extracellular DNA and dead cells) and Calcofluor White D (D, hyphae) signals. ECM deposition (red fluorescence) is observed mainly in those areas where the hyphal network is more compact and highly interwoven (blue fluorescence).

SUPPLEMENTARY FIGURE S2 Standard curves obtained relating the log of each known concentration of gene copy number and the corresponding value of threshold cycle for mono-specific fungal **(A)** or bacterial **(B)** biofilms The values of slope and primers efficiency are shown to indicate the goodness of fit of the calibration curves.

SUPPLEMENTARY FIGURE S3 Linear regression curves between gene copy number and dry weight of biomass in monospecific biofilms. **(A)** 18S rRNA gene copy number *vs Pleurotus ostreatus* biomass. **(B)** 16S rRNA gene copy number *vs. Pseudomonas alcaliphila* biomass.

## References

[ref1] Banitz T. Johst K. Wick L. Y. Schamfuß S. Harms H. Frank K. (2013). Highways versus pipelines: contributions of two fungal transport mechanisms to efficient bioremediation. Environ. Microbiol. Rep. 5, 211–218. 10.1111/1758-2229.12002 23584964

[ref2] Bawadekji A. Mridha M. A. U. Al Ali M. Jamith Basha W. J. (2017). Antimicrobial activities of oyster mushroom *Pleurotus ostreatus* (Jacq. ex. Fr.) Kummer. J. Appl. Environ. Biol. Sci. 7, 227–231.

[ref3] Boles B. R. Singh P. K. (2008). Endogenous oxidative stress produces diversity and adaptability in biofilm communities. Proc. Natl. Acad. Sci. 105, 12503–12508. 10.1073/pnas.0801499105 18719125 PMC2527941

[ref4] Boudarel H. Mathias J. D. Blaysat B. Grédiac M. (2018). Towards standardized mechanical characterization of microbial biofilms: analysis and critical review. NPJ Biofilms Microbiomes 4:17. 10.1038/s41522-018-0062-5, PMID: 30131867 PMC6102240

[ref5] Branda S. S. Vik S. Friedman L. Kolter R. (2005). Biofilms: the matrix revisited. Trends Microbiol. 13, 20–26. 10.1016/j.tim.2004.11.006, PMID: 15639628

[ref6] Ceri H. Olson M. E. Stremick C. Read R. R. Morck D. Buret A. (1999). The Calgary biofilm device: new technology for rapid determination of antibiotic susceptibilities of bacterial biofilms. J. Clin. Microbiol. 37, 1771–1776. PMID: 10325322 10.1128/jcm.37.6.1771-1776.1999 PMC84946

[ref7] Chandra J. Kuhn D. M. Mukherjee P. K. Hoyer L. L. McCormick T. Ghannoum M. A. (2001). Biofilm formation by the fungal pathogen *Candida albicans*: development, architecture, and drug resistance. J. Bacteriol. 183, 5385–5394. 10.1128/jb.183.18.5385-5394.2001 11514524 PMC95423

[ref8] Cho Y. S. Kim J. S. Crowley D. E. Cho B. G. (2003). Growth promotion of the edible fungus *Pleurotus ostreatus* by fluorescent pseudomonads. FEMS Microbiol. Lett. 218, 271–276. 10.1016/S0378-1097(02)01144-8 12586403

[ref9] Covino S. Stella T. D’Annibale A. Lladó S. Baldrian P. Čvančarová M. (2016). Comparative assessment of fungal augmentation treatments of a fine-textured and historically oil-contaminated soil. Sci. Total Environ. 566, 250–259. 10.1016/j.scitotenv.2016.05.018 27220102

[ref10] de Rossi B. P. García C. Alcaraz E. Franco M. (2014). *Stenotrophomonas maltophilia* interferes via the DSF-mediated quorum sensing system with *Candida albicans* filamentation and its planktonic and biofilm modes of growth. Rev. Argent. Microbiol. 46, 288–297. 10.1016/S0325-7541(14)70084-7, PMID: 25576410

[ref11] Deveau A. Bonito G. Uehling J. Paoletti M. Becker M. Bindschedler S.. (2018). Bacterial-fungal interactions: ecology, mechanisms and challenges. FEMS Microbiol. Rev. 42, 335–352. 10.1093/femsre/fuy008, PMID: 29471481

[ref12] Di Bonaventura G. Pompilio A. Picciani C. Iezzi M. D’Antonio D. Piccolomini R. (2006). Biofilm formation by the emerging fungal pathogen *Trichosporon asahii*: development, architecture, and antifungal resistance. Antimicrob. Agents Chemother. 50, 3269–3276. 10.1128/AAC.00556-06, PMID: 17005804 PMC1610057

[ref13] Domenech M. Pedrero-Vega E. Prieto A. García E. (2016). Evidence of the presence of nucleic acids and β-glucan in the matrix of non-typeable *Haemophilus influenzae* in vitro biofilms. Sci. Rep. 6:36424. 10.1038/srep36424 27805043 PMC5090351

[ref14] Essig A. Hofmann D. Münch D. Gayathri S. Künzler M. Kallio P. T.. (2014). Copsin, a novel peptide-based fungal antibiotic interfering with the peptidoglycan synthesis. J. Biol. Chem. 289, 34953–34964. 10.1074/jbc.M114.599878, PMID: 25342741 PMC4263892

[ref15] Farrell F. D. Gralka M. Hallatschek O. Waclaw B. (2017). Mechanical interactions in bacterial colonies and the surfing probability of beneficial mutations. J. R. Soc. Interf. 14:20170073. 10.1098/rsif.2017.0073 PMC5493792 28592660

[ref16] Ferrera I. Massana R. Balagué V. Pedrós-Alió C. Sánchez O. Mas J. (2010). Evaluation of DNA extraction methods from complex phototrophic biofilms. Biofouling 26, 349–357. 10.1080/08927011003605870, PMID: 20140796

[ref17] Finkel J. S. Mitchell A. P. (2011). Genetic control of *Candida albicans* biofilm development. Nat. Rev. Microbiol. 9, 109–118. 10.1038/nrmicro2475 21189476 PMC3891587

[ref18] Fredricks D. N. Smith C. Meier A. (2005). Comparison of six DNA extraction methods for recovery of fungal DNA as assessed by quantitative PCR. J. Clin. Microbiol. 43, 5122–5128. 10.1128/JCM.43.10.5122-5128.2005, PMID: 16207973 PMC1248488

[ref19] Frostegård Å. Tunlid A. Bååth E. (2011). Use and misuse of PLFA measurements in soils. Soil Biol. Biochem. 43, 1621–1625. 10.1016/j.soilbio.2010.11.021

[ref20] Gingichashvili S. Duanis-Assaf D. Shemesh M. Featherstone J. D. Feuerstein O. Steinberg D. (2017). *Bacillus subtilis* biofilm development–a computerized study of morphology and kinetics. Front. Microbiol. 8:2072. 10.3389/fmicb.2017.02072, PMID: 29163384 PMC5674941

[ref21] Goodwine J. Gil J. Doiron A. Valdes J. Solis M. Higa A.. (2019). Pyruvate-depleting conditions induce biofilm dispersion and enhance the efficacy of antibiotics in killing biofilms in vitro and in vivo. Sci. Rep. 9:3763. 10.1038/s41598-019-40378-z, PMID: 30842579 PMC6403282

[ref22] Guennoc C. M. Rose C. Guinnet F. Miquel I. Labbé J. Deveau A. (2017). A new method for qualitative multi-scale analysis of bacterial biofilms on filamentous fungal colonies using confocal and electron microscopy. J. Vis. Exp. 119:e54771. 10.3791/54771 PMC5352296 28190036

[ref23] Guennoc C. M. Rose C. Labbé J. Deveau A. (2018). Bacterial biofilm formation on the hyphae of ectomycorrhizal fungi: a widespread ability under controls? FEMS Microbiol. Ecol. 94:fiy093. 10.1093/femsec/fiy093 29788056

[ref24] Harding M. W. Marques L. L. Howard R. J. Olson M. E. (2009). Can filamentous fungi form biofilms? Trends Microbiol. 17, 475–480. 10.1016/j.tim.2009.08.007 19833519

[ref25] Hearst R. Nelson D. McCollum G. Millar B. C. Maeda Y. Goldsmith C. E. (2009). An examination of antibacterial and antifungal properties of constituents of shiitake (*Lentinula edodes*) and oyster (*Pleurotus ostreatus*) mushrooms. Compl. Ther. Clin. Pract. 15, 5–7. 10.1016/j.ctcp.2008.10.002 19161947

[ref26] Herath H. M. L. I. Upamali A. Vithanage M. Seneviratne G. (2014). Developed fungal–bacterial biofilms as a novel tool for bioremoval of hexavalent chromium from wastewater. Chem. Ecol. 30, 418–427. 10.1080/02757540.2013.861828

[ref27] Hogan D. A. Kolter R. (2002). *Pseudomonas-Candida* interactions: an ecological role for virulence factors. Science 296, 2229–2232. 10.1126/science.1070784, PMID: 12077418

[ref28] Hogan D. A. Vik A. Kolter R. (2004). A *Pseudomonas aeruginosa* quorum-sensing molecule influences *Candida albicans* morphology. Mol. Microbiol. 54, 1212–1223. 10.1111/j.1365-2958.2004.04349.x, PMID: 15554963

[ref29] Holmer L. Stenlid J. (1993). The importance of inoculum size for the competitive ability of wood decomposing fungi. FEMS Microbiol. Ecol. 12, 169–176. 10.1111/j.1574-6941.1993.tb00029.x

[ref30] Hover T. Maya T. Ron S. Sandovsky H. Shadkchan Y. Kijner N. (2016). Mechanisms of bacterial (*Serratia marcescens*) attachment to, migration along, and killing of fungal hyphae. Appl. Environ. Microbiol. 82, 2585–2594. 10.1128/AEM.04070-15 26896140 PMC4836416

[ref31] Hunt S. M. Werner E. M. Huang B. Hamilton M. A. Stewart P. S. (2004). Hypothesis for the role of nutrient starvation in biofilm detachment. Appl. Environ. Microbiol. 70, 7418–7425. 10.1128/AEM.70.12.7418-7425.2004 15574944 PMC535154

[ref32] Ibáñez de Aldecoa A. L. Zafra O. González-Pastor J. E. (2017). Mechanisms and regulation of extracellular DNA release and its biological roles in microbial communities. Front. Microbiol. 8:1390. 10.3389/fmicb.2017.01390 28798731 PMC5527159

[ref33] Jayasinghearachchi H. S. Seneviratne G. (2004). Can mushrooms fix atmospheric nitrogen? J. Biosci. 29, 293–296. 10.1007/BF02702611 15381850

[ref34] Jayasinghearachchi H. S. Seneviratne G. (2006a). A mushroom-fungus helps improve endophytic colonization of tomato by *Pseudomonas fluorescens* through biofilm formation. Res. J. Microbiol. 1, 83–89. 10.3923/jm.2006.83.89

[ref35] Jayasinghearachchi H. S. Seneviratne G. (2006b). Fungal solubilization of rock phosphate is enhanced by forming fungal-rhizobial biofilms. Soil Biol. Biochem. 38, 405–408. 10.1016/j.soilbio.2005.06.004

[ref36] Kataky R. Knowles E. (2018). Biofilm formation on abiotic surfaces and their redox activity. Curr. Opin. Electrochem. 12, 121–128. 10.1016/j.coelec.2018.07.007

[ref37] Křesinová Z. Linhartová L. Filipová A. Ezechiáš M. Mašín P. Cajthaml T. (2018). Biodegradation of endocrine disruptors in urban wastewater using *Pleurotus ostreatus* bioreactor. New Biotechnol. 43, 53–61. 10.1016/j.nbt.2017.05.004, PMID: 28502780

[ref38] Lemos M. Mergulhão F. Melo L. Simões M. (2015). The effect of shear stress on the formation and removal of *Bacillus cereus* biofilms. Food Bioprod. Process. 93, 242–248. 10.1016/j.fbp.2014.09.005

[ref39] Leonardi V. Giubilei M. A. Federici E. Spaccapelo R. Šašek V. Novotny C.. (2008). Mobilizing agents enhance fungal degradation of polycyclic aromatic hydrocarbons and affect diversity of indigenous bacteria in soil. Biotechnol. Bioeng. 101, 273–285. 10.1002/bit.21909, PMID: 18727031

[ref40] Liu W. Røder H. L. Madsen J. S. Bjarnsholt T. Sørensen S. J. Burmølle M. (2016). Interspecific bacterial interactions are reflected in multispecies biofilm spatial organization. Front. Microbiol. 7:1366. 10.3389/fmicb.2016.01366 27630624 PMC5005372

[ref41] Lopes S. P. Azevedo N. F. Pereira M. O. (2018). Quantitative assessment of individual populations within polymicrobial biofilms. Sci. Rep. 8:9494. 10.1038/s41598-018-27497-9, PMID: 29934504 PMC6015014

[ref42] Makovcova J. Babak V. Kulich P. Masek J. Slany M. Cincarova L. (2017). Dynamics of mono- and dual-species biofilm formation and interactions between *Staphylococcus aureus* and gram-negative bacteria. Microb. Biotechnol. 10, 819–832. 10.1111/1751-7915.12705, PMID: 28401747 PMC5481519

[ref43] McKew B. A. Taylor J. D. McGenity T. J. Underwood G. J. (2011). Resistance and resilience of benthic biofilm communities from a temperate saltmarsh to desiccation and rewetting. ISME J. 5, 30–41. 10.1038/ismej.2010.91, PMID: 20596071 PMC3105671

[ref44] Melloul E. Luiggi S. Anaïs L. Arné P. Costa J. M. Fihman V.. (2016). Characteristics of *Aspergillus fumigatus* in association with *Stenotrophomonas maltophilia* in an in vitro model of mixed biofilm. PLoS One 11:e0166325. 10.1371/journal.pone.0166325, PMID: 27870863 PMC5117647

[ref45] Melucci D. Fedi S. Locatelli M. Locatelli C. Montalbani S. Cappelletti M. (2013). Application of pyrolysis-gas chromatography-mass spectrometry and multivariate analysis to study bacteria and fungi in biofilms used for bioremediation. Curr. Drug Targets 14, 1023–1033. 10.2174/1389450111314090011, PMID: 23721185

[ref46] Mensah-Attipoe J. Reponen T. Veijalainen A. M. Rintala H. Täubel M. Rantakokko P.. (2016). Comparison of methods for assessing temporal variation of growth of fungi on building materials. Microbiology 162, 1895–1903. 10.1099/mic.0.000372, PMID: 27655355

[ref47] Miller D. N. Bryant J. E. Madsen E. L. Ghiorse W. C. (1999). Evaluation and optimization of DNA extraction and purification procedures for soil and sediment samples. Appl. Environ. Microbial. 65, 4715–4724. 10.1128/aem.65.11.4715-4724.1999 PMC91634 10543776

[ref48] Mitchell K. F. Zarnowski R. Andes D. R. (2016). “The extracellular matrix of fungal biofilms” in Fungal biofilms and related infections. ed. Imbert C. (Switzerland: Springer International Publishing), 21–35. 10.1007/5584_2016_6 27271680

[ref49] Nadkarni M. A. Martin F. E. Jacques N. A. Hunter N. (2002). Determination of bacterial load by real-time PCR using a broad-range (universal) probe and primers set. Microbiology 148, 257–266. 10.1099/00221287-148-1-257, PMID: 11782518

[ref50] Oates L. G. Read H. W. Gutknecht J. L. Duncan D. S. Balser T. B. Jackson R. D. (2017). A lipid extraction and analysis method for characterizing soil microbes in experiments with many samples. JoVE 125:e55310. 10.3791/55310 PMC5553326 28745639

[ref51] Oh E. Jeon B. (2014). Role of alkyl hydroperoxide reductase (AhpC) in the biofilm formation of *Campylobacter jejuni*. PLoS One 9:e87312. 10.1371/journal.pone.0087312, PMID: 24498070 PMC3909096

[ref52] Parahitiyawa N. B. Samaranayake Y. H. Samaranayake L. P. Ye J. Tsang P. W. K. Cheung B. P. K. (2006). Interspecies variation in *Candida* biofilm formation studied using the Calgary biofilm device. APMIS 114, 298–306. 10.1111/j.1600-0463.2006.apm_394.x 16689830

[ref53] Pereira-Cenci T. Deng D. M. Kraneveld E. A. Manders E. M. M. Cury A. A. D. B. Ten Cate J. M.. (2008). The effect of *Streptococcus mutans* and *Candida glabrata* on *Candida albicans* biofilms formed on different surfaces. Arch. Oral Biol. 53, 755–764. 10.1016/j.archoralbio.2008.02.015, PMID: 18395698

[ref54] Pesciaroli L. Petruccioli M. Federici F. D’Annibale A. (2013a). *Pleurotus ostreatus* biofilms exhibit higher tolerance to toxicants than free-floating counterparts. Biofouling 29, 1043–1055. 10.1080/08927014.2013.825901 23998200

[ref55] Pesciaroli L. Petruccioli M. Federici F. D’Annibale A. (2013c). *Pleurotus ostreatus* biofilm-forming ability and ultrastructure are significantly influenced by growth medium and support type. J. Appl. Microbiol. 114, 1750–1762. 10.1111/jam.12170 23414514

[ref56] Pesciaroli L. Petruccioli M. Fedi S. Firrincieli A. Federici F. D’Annibale A. (2013b). Characterization of *Pleurotus ostreatus* biofilms by using the Calgary biofilm device. Appl. Environ. Microbiol. 79, 6083–6092. 10.1128/AEM.02099-13 23892744 PMC3811380

[ref57] Peters B. M. Jabra-Rizk M. A. Scheper M. A. Leid J. G. Costerton J. W. Shirtliff M. E. (2010). Microbial interactions and differential protein expression in *Staphylococcus aureus*-*Candida albicans* dual-species biofilms. FEMS Immunol. Med. Microbiol. 59, 493–503. 10.1111/j.1574-695X.2010.00710.x, PMID: 20608978 PMC2936118

[ref58] Pierce C. G. Uppuluri P. Tristan A. R. Wormley F. L. Mowat E. Ramage G. (2008). A simple and reproducible 96-well plate-based method for the formation of fungal biofilms and its application to antifungal susceptibility testing. Nat. Protoc. 3, 1494–1500. 10.1038/nport.2008.141 18772877 PMC2741160

[ref59] Pitt J. I. (1984). Significance of potentially toxigenic fungi in foods. Food Technol. Australia 36, 218–219.

[ref60] Ridl J. Suman J. Fraraccio S. Hradilova M. Strejcek M. Cajthaml T. (2018). Complete genome sequence of *Pseudomonas alcaliphila* JAB1 (=DSM 26533), a versatile degrader of organic pollutants. Stand. Genom. Sci. 13:3. 10.1186/s40793-017-0306-7 PMC5796565 29435100

[ref61] Ritz K. Young I. M. (2004). Interactions between soil structure and fungi. Mycologist 18, 52–59. 10.1017/S0269-915X(04)00201-0

[ref62] Rivardo F. Turner R. J. Allegrone G. Ceri H. Martinotti M. G. (2009). Anti-adhesion activity of two biosurfactants produced by *Bacillus* spp. prevents biofilm formation of human bacterial pathogens. Appl. Microbiol. Biotechnol. 83, 541–553. 10.1007/s00253-009-1987-7, PMID: 19343338

[ref63] Ryder V. J. Chopra I. O’Neill A. J. (2012). Increased mutability of staphylococci in biofilms as a consequence of oxidative stress. PLoS One 7:e47695. 10.1371/journal.pone.0047695, PMID: 23110091 PMC3480535

[ref64] Santopolo L. Marchi E. Decorosi F. Galardini M. Brilli M. Giovannetti L. (2013). Draft genome sequence of chromate-resistant and biofilm-producing strain *Pseudomonas alcaliphila* 34. Genome Announc. 1, e00125–e00112. 10.1128/genomeA.00125-12 PMC3587930 23469336

[ref65] Santopolo L. Marchi E. Frediani L. Decorosi F. Viti C. Giovannetti L. (2012). A novel approach combining the Calgary biofilm device and phenotype microArray for the characterization of the chemical sensitivity of bacterial biofilms. Biofouling 28, 1023–1032. 10.1080/08927014.2012.726352 23004019

[ref66] Schnürer J. (1993). Comparison of methods for estimating the biomass of three food-borne fungi with different growth patterns. Appl. Environ. Microbiol. 59, 552–555. PMID: 8434921 10.1128/aem.59.2.552-555.1993 PMC202142

[ref67] Schutter M. E. Dick R. P. (2000). Comparison of fatty acid methyl ester (FAME) methods for characterizing microbial communities. Soil Sci. Soc. Am. J. 64, 1659–1668. 10.2136/sssaj2000.6451659x

[ref68] Seneviratne G. Zavahir J. S. Bandara M. L. M. A. Weerasekara W. M. M. S. (2008). Fungal-bacterial biofilms: their development for novel biotechnological applications. World J. Microbiol. Biotechnol. 24, 739–743. 10.1007/s11274-007-9539-8

[ref69] Singh R. Paul D. Jain R. K. (2006). Biofilms: implications in bioremediation. Trends Microbiol. 14, 389–397. 10.1016/j.tim.2006.07.001 16857359

[ref70] Smith W. P. Davit Y. Osborne J. M. Kim W. Foster K. R. Pitt-Francis J. M. (2017). Cell morphology drives spatial patterning in microbial communities. Proc. Natl. Acad. Sci. 114, E280–E286. 10.1073/pnas.1613007114, PMID: 28039436 PMC5255625

[ref71] Stazi S. R. Moscatelli M. C. Papp R. Crognale S. Grego S. Martin M. (2017). A multi-biological assay approach to assess microbial diversity in arsenic (as) contaminated soils. Geomicrobiol J. 34, 183–192. 10.1080/01490451.2016.1189015

[ref72] Stewart P. S. Franklin M. J. (2008). Physiological heterogeneity in biofilms. Nat. Rev. Microbiol. 6, 199–210. 10.1038/nrmicro1838, PMID: 18264116

[ref73] Stöckli M. Lin C. W. Sieber R. Plaza D. F. Ohm R. A. Künzler M. (2017). *Coprinopsis cinerea* intracellular lactonases hydrolyze quorum sensing molecules of gram-negative bacteria. Fungal Genet. Biol. 102, 49–62. 10.1016/j.fgb.2016.07.009, PMID: 27475110

[ref74] Sztajer H. Szafranski S. P. Tomasch J. Reck M. Nimtz M. Rohde M.. (2014). Cross-feeding and interkingdom communication in dual-species biofilms of *Streptococcus mutans* and *Candida albicans*. ISME J. 8, 2256–2271. 10.1038/ismej.2014.73, PMID: 24824668 PMC4992082

[ref75] Vainio E. J. Hantula J. (2000). Direct analysis of wood-inhabiting fungi using denaturing gradient gel electrophoresis of amplified ribosomal DNA. Mycol. Res. 104, 927–936. 10.1017/S0953756200002471

[ref76] Wilking J. N. Zaburdaev V. De Volder M. Losick R. Brenner M. P. Weitz D. A. (2013). Liquid transport facilitated by channels in *Bacillus subtilis* biofilms. Proc. Natl. Acad. Sci. 110, 848–852. 10.1073/pnas.1216376110 23271809 PMC3549102

[ref77] Wolfmeier H. Pletzer D. Mansour S. C. Hancock R. E. (2017). New perspectives in biofilm eradication. ACS Infect. Diseases 4, 93–106. 10.1021/acsinfecdis.7b00170 29280609

[ref78] Woodward S. Boddy L. (2008). “Interactions between saprotrophic fungi” in British mycological society Symposia series. Vol. 28, eds. Boddy L. Frankland J. C. van West P. (Academic Press), 125–141.

[ref79] Zago C. E. Silva S. Sanitá P. V. Barbugli P. A. Dias C. M. I. Lordello V. B.. (2015). Dynamics of biofilm formation and the interaction between *Candida albicans* and methicillin-susceptible (MSSA) and-resistant *Staphylococcus aureus* (MRSA). PLoS One 10:e0123206. 10.1371/journal.pone.0123206, PMID: 25875834 PMC4395328

