# Supplementary figures and images for: Time-Dependent Changes in Morphostructural Properties and Relative Abundances of Contributors in Pleurotus ostreatus/Pseudomonas alcaliphila Mixed Biofilms
Source: Front Microbiol. 2019 Aug 9;10:1819. doi: 10.3389/fmicb.2019.01819 (PMC6695841; doi:10.3389/fmicb.2019.01819)

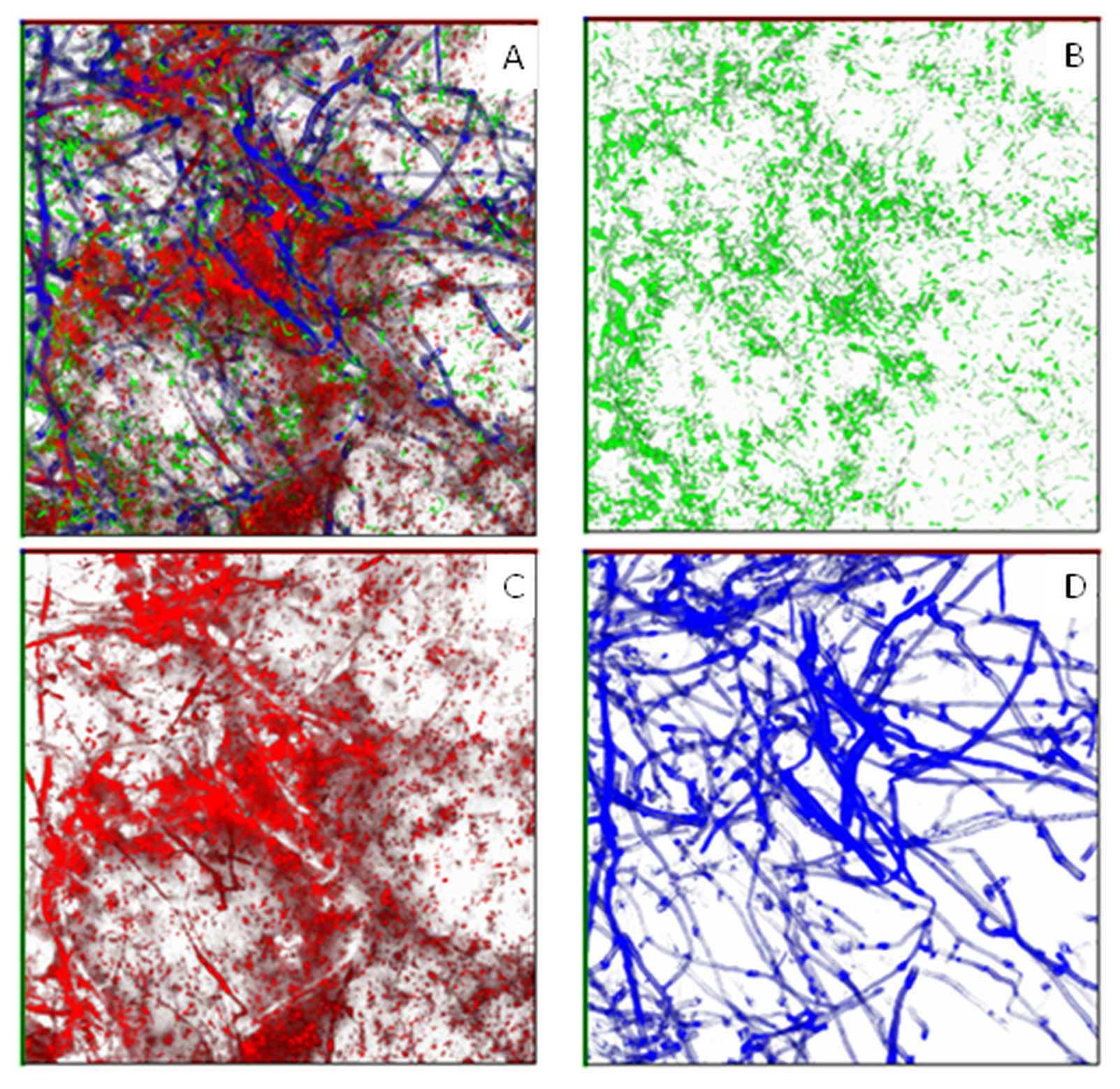

Supplement: SUPPLEMENTARY FIGURE S1 — Volume rendering of P. alcaliphila and P. ostreatus 48-h-old mixed biofilm. Image A is the overlay of the SYTO 9 (B, live bacterial cells), PI (C, extracellular DNA and dead cells) and Calcofluor White D (D, hyphae) signals. ECM deposition (red fluorescence) is observed mainly in those areas where the hyphal network is more compact and highly interwoven (blue fluorescence). [file Image_1.TIF]

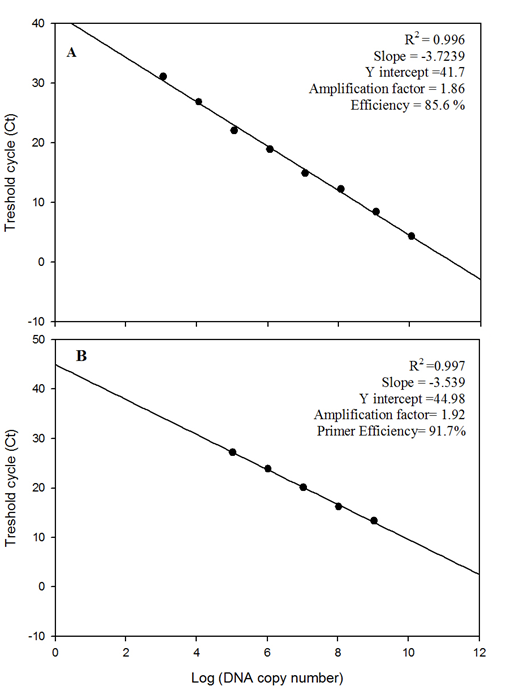

Supplement: SUPPLEMENTARY FIGURE S2 — Standard curves obtained relating the log of each known concentration of gene copy number and the corresponding value of threshold cycle for mono-specific fungal (A) or bacterial (B) biofilms The values of slope and primers efficiency are shown to indicate the goodness of fit of the calibration curves. [file Image_2.TIF]

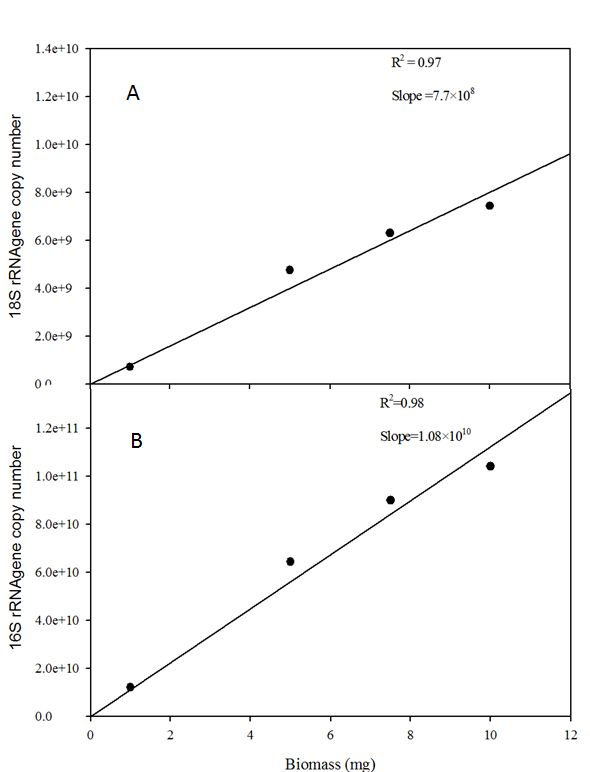

Supplement: SUPPLEMENTARY FIGURE S3 — Linear regression curves between gene copy number and dry weight of biomass in monospecific biofilms. (A) 18S rRNA gene copy number vs Pleurotus ostreatus biomass. (B) 16S rRNA gene copy number vs. Pseudomonas alcaliphila biomass. [file Image_3.TIF]
